# Pomegranate Byproduct Extracts as Ingredients for Producing Experimental Cheese with Enhanced Microbiological, Functional, and Physical Characteristics

**DOI:** 10.3390/foods10112669

**Published:** 2021-11-03

**Authors:** Lucia Parafati, Fabiola Pesce, Laura Siracusa, Biagio Fallico, Cristina Restuccia, Rosa Palmeri

**Affiliations:** 1 Di3A, Dipartimento di Agricoltura, Alimentazione e Ambiente, University of Catania, via S. Sofia 100, 95123 Catania, Italy; lucia.parafati@unict.it (L.P.); fabiolapesce3@gmail.com (F.P.); bfallico@unict.it (B.F.); rpalmeri@unict.it (R.P.); 2 CNR-ICB, Consiglio Nazionale delle Ricerche-Istituto di Chimica Biomolecolare, via Paolo Gaifami 18, 95126 Catania, Italy; laura.siracusa@icb.cnr.it

**Keywords:** bacterial food pathogens, *Staphylococcus aureus*, byproducts reuse, polyphenol profile, antioxidant compounds

## Abstract

Pomegranate peel and mesocarp, considered as wastes of fruit processing, are rich sources of beneficial phytochemicals, including hydrolyzable tannins and flavonoids, with proven antimicrobial and antioxidant activity, which can be employed for improving the overall quality of food products. In the present study, extracts from pomegranate peel (PPW) and mesocarp (PMW) were obtained through a water extraction method and evaluated for in vitro antimicrobial activity and polyphenol content. The two extracts were then added during the cheese-making process in order to create a new functional cheese with improved microbiological and physico-chemical characteristics. Antimicrobial in vitro assays evidenced a substantial efficacy of both extracts against *Staphylococcus aureus*, which often causes staphylococcal food poisoning outbreaks linked to the consumption of raw milk cheeses and artisanal cheeses. For this reason, a simulated cheese contamination was carried out in order to assess if pomegranate extracts can exert antimicrobial activity towards this pathogen even when incorporated into the cheese matrix. Milk enriched with pomegranate extracts (PPW and PMW) was used to produce two different experimental cheeses, which were then evaluated for yield, polyphenol content, and microbiological as well as physico-chemical traits throughout the refrigerated storage. Despite the low concentration of the extracts, the treated cheeses showed an increase in firmness and a slight decrease in *S. aureus* counts, of more than one log unit in comparison to the control cheese, for up to 12 d of cold storage. Such results support the reuse of agro-food byproducts, in substitution to chemical food preservatives, as the key to a circular economy.

## 1. Introduction

Artisanal cheese is often produced using raw milk, which could represent a vehicle of a broad spectrum of food pathogens such as *Listeria monocytogenes, Escherichia coli*, *Salmonella* spp., and *Staphylococcus aureus*. The latter, in particular, is considered one of the most hazardous, since several staphylococcal food poisoning outbreaks have been associated with the consumption of raw milk cheeses and artisanal cheese production [1]. In addition, dairy processing equipment and environments, as well as food handlers, could represent sources for the introduction of *S. aureus* into the dairy product supply chain [2]. Therefore, the development of strategies for the control of this pathogenic species are necessary. According to the European Commission Regulation (EC) No. 1333/2008 [3] on food additives, preservatives are defined as “substances which prolong the shelf-life of foods by protecting them against deterioration caused by micro-organisms and/or which protect against growth of pathogenic micro-organisms”. The Commission Regulation (EU) No. 1129/2011 [4], amending Annex II to Regulation (EC) No. 1333/2008, establishes a Union list of food additives for unripened and ripened cheese. These preservatives are considered safe for human health in the allowed dosage, but they are largely used in the food industry, increasing their real daily consumption and thus causing health problems [5]. Therefore, worry about the safety of some chemical additives and consumers’ distrust towards them has steered to a rising interest in natural preservatives, such as plant-based compounds.

In recent years, several scientific works [6,7,8] have reviewed the latest scientific findings on the use and effects of herbal extracts in the dairy sector, showing the beneficial effects of the addition of natural compounds into different formulations. Moreover, the use of plant extracts and essential oils in cheese making could confer functional properties to the final dairy products [9,10,11].

Fruit byproducts are a source of phenolic compounds, possessing antimicrobial and antioxidant activities, which can be converted into high-value-added compounds for food, cosmetic, and pharmaceutical products with a positive impact on sustainability and economy indicators of the fruit chain. Pomegranate (*Punica granatum* L.) fruit processing, for obtaining juice or ready-to-eat arils, generates a huge amount of byproducts, mainly consisting of the outer leathery skin, commonly called peel, and mesocarp, which account for at least 50% of the whole fruit. The reuse of the aforementioned byproducts would also generate an economic advantage for companies that generally have to dispose large quantities of waste, becoming a resource instead.

Pomegranate may be identified as one of the most studied fruit matrices of the last decade. The growing interest of the scientific community towards this product is not limited to juice or arils, which represent the edible part, but is also extended to its byproducts, mainly the peel [12,13,14]. Seeds, mesocarp, and other waste matrices obtained from pomegranate juice production have also been extensively investigated [15,16].

The extracts from pomegranate byproducts have been shown to possess antimicrobial activity against food pathogenic fungi and bacteria, including *S. aureus* [17,18,19,20,21]. In addition to their antioxidant activity, pomegranate peel extracts have also been investigated as antimicrobials in various studies because of their many bioactive and nutritional components [22]. However, such an antimicrobial effect is strictly correlated to the part of pomegranate used (peel, flower, leaf, stem, or fruit pericarp) and to the type of solvent used in extraction (acetonic, methanolic, water, or ethanolic). As regards the latter, obviously not all the extracts obtained using solvents can then be applied to foods, even if they are eliminated, limiting the use of permitted extraction solvents [23].

In regard to dairy products, recent studies aimed to evaluate the use of pomegranate juice and peel extracts, with the purpose to improve the content of bioactive compounds of the products.

With a particular reference to cheese, only two recent works evaluated the use of pomegranate peel extract and powered peel for improving the antioxidant stability of a fermented milk product (curd) and for increasing the lipid oxidative stability as well as the storage quality of an Indian cheese called “Kalari”, respectively [24,25]. In both studies, pomegranate extracts were added to the cheese after coagulation, so it remains unknown as to how phenolic compounds of pomegranate can affect the cheese-making process and which phenolic compound can bind with the curd. The development of an improved cheese product, by adding a functional ingredient to milk that can positively affect the cheese-making process and the microbial stability of the final product, could represent an important innovation for the food industry. Therefore, the present study aims at evaluating the effects of the use of two pomegranate byproduct water extracts on the cheese-making process, in order to create a new functional cheese with improved microbiological and physico-chemical characteristics. The principal objectives of this study were (i) to investigate the in vitro and in-cheese antimicrobial activity of two extracts made from pomegranate peel and mesocarp, (ii) to determine the retained fraction of polyphenolic compounds on the cheese matrix, and (iii) to evaluate the physico-chemical properties of cheese containing the pomegranate extracts.

## 2. Materials and Methods

### 2.1. Preparation of Pomegranate Byproduct Extracts

Ripe Sicilian pomegranate (*Punica granatum*) fruits of the cultivar “Wonderful”, obtained from a local consortium of producers (Consorzio “Kore” Frutti di Sicilia, https://www.consorziokore.it/en/, accessed on 4 September 2021), were accurately washed and dried with sorbent paper, and then manually divided into leathery peel (exocarp), fleshy mesocarp, and arils. Aliquots of fresh peel and mesocarp (ca.100 g each) were separately finely chopped, put in a laboratory flask and suspended in sterile distilled water (500 mL). The resulting suspensions were left stirring (5 rpm) on a magnetic plate overnight in the dark at room temperature, then filtered under a vacuum on a Buchner funnel equipped with moistened standard laboratory filter paper (Whatman Grade 3, Merck Italia, Milan, Italy) to recover the mother solutions, which presented pH values of 3.52 and 3.50 for pomegranate peel and mesocarp, respectively. Aliquots (2 mL) of these solutions were opportunely diluted to obtain concentrations of 5–12 mg fresh material/mL that were put in HPLC 2 mL amber vials and immediately analyzed (see next paragraph). The remaining part of the solutions coming from the extractions were lyophilized (Lyoquest-85, Telstar Italy, Legnano, Milan, Italy) and then stored in a dark and dry place until use. This procedure was repeated trice to verify its reproducibility in terms of composition and yield; the results are given as the mean value and are the following: pomegranate peel water extract (PPW), 11.8 g (11.8% yield from fresh vegetable material); pomegranate mesocarp water extract (PMW), 12.6 g (12.6% yield from fresh vegetable material).

### 2.2. HPLC/DAD and HPLC/ESI-MS Analyses

HPLC-grade solvents (water and acetonitrile) were purchased from VWR (Milan, Italy) and high-purity commercial analytical standards were obtained from Sigma-Aldrich (Milan, Italy). Chromatographic analyses were carried out on an Ultimate3000 UHPLC-focused instrument equipped with a binary high-pressure pump, a Photodiode Array Detector, a Thermostatted Column Compartment, and an Automated Sample Injector (Thermo Fisher Scientific, Inc., Milan, Italy). Analytical runs were performed using the same chromatographic conditions (column elution program and solvents) reported by Russo et al. (2021) [26]. Quantification was carried out at 280 nm for gallic acid and its derivatives (including HHDP derivatives, pedunculagins, and granatins) using gallic acid as a reference (r^2^ = 0.9999). Similarly, quantification of ellagitannins was made at 360 nm using punicalagin (r^2^ = 0.9998) as a reference, while ellagic acid and its derivatives were quantified at the same wavelength using ellagic acid (r^2^ = 0.9997) as an external standard. In order to unambiguously identify the chromatographic signals, HPLC/ESI-MS analyses were also performed. The HPLC apparatus used was as described above, whilst ESI mass spectra were acquired using the same instrumentation, operating conditions, and acquisition software as previously described [18]. All analyses were carried out in triplicate.

### 2.3. In Vitro Antimicrobial Activity of Pomegranate Extracts

#### 2.3.1. Agar Well Diffusion Assay

The two freeze-dried pomegranate extracts (PPW and PMW) were individually solved in sterile distilled water to a final concentration of 1 g/100 mL (*w/v*). Each solution was filter-sterilized using a 0.20 µm pore size membrane filter (Millipore^®^, Burlington, MA, USA) and then used for the subsequent in vitro antibacterial activity assay.

The antimicrobial activity of each extract was evaluated by the agar well diffusion test against the following bacterial species: *Escherichia coli*, *Listeria innocua*, *Staphylococcus aureus*, *S. haemolyticus*, *Salmonella enterica*, *Bacillus subtilis*, *B. cereus*, and *Pseudomonas fluorescens*, belonging to the Di3A (Dipartimento di Agricoltura, Alimentazione e Ambiente, University of Catania, Catania, Italy) collection. Stock cultures were routinely maintained at 4 °C on Petri dishes containing Nutrient Agar (NA, Oxoid, Basingstoke, UK).

Twenty-four h bacterial cultures, grown in Nutrient Broth (NB, Oxoid, Basingstoke, UK), were individually inoculated into 20 mL autoclaved and cooled, at 45 °C Nutrient Agar (NA, Oxoid, Basingstoke, UK), to obtain a final cell concentration of 10^6^/mL, rapidly vortexed, and poured into sterile Petri plates (diameter of 90 mm). After solidification, wells were made into agar plates by using a sterile cork borer (5 mm diameter) and filled with 80 μL of each extract. The plates inoculated with the above-mentioned bacteria were incubated at 35 °C, with the exception of *P. fluorescens*, which was incubated at 27 °C. In the control plates the wells were filled only with sterile distilled water.

The inhibitory effect of each extract was assessed after 24–48 h of incubation, by measuring the size (cm) of the inhibition zone (no bacterial growth) around the well. Each test was performed in triplicate.

#### 2.3.2. Minimal Inhibitory Concentration (MIC) and Minimal Bactericidal Concentration (MBC) towards *S. aureus* Strain

Among the bacterial strains sensitive to the pomegranate extracts, *S. aureus* has been chosen for further investigation, since this species is considered one of the most hazardous for the consumption of raw milk cheeses and artisanal cheeses. The minimal inhibitory concentration (MIC) and minimal bactericidal concentration (MBC) of PPW and PMW were determined in a liquid medium.

*S. aureus* was allowed to grow for 24 h at 35 °C with shaking. After 24 h the cells were collected by centrifugation and re-suspended in sterile NB in order to obtain a stock culture of 10^9^ CFU/mL. Tubes containing 5 mL of NB with decreasing concentrations (100, 50, 25, 12, 5, 2.5, 1.2, 0.5, and 0.1 mg/mL) of PPW and PMW were inoculated with the bacterial stock culture in order to obtain, in the medium, a final concentration of 10^6^ CFU/mL of *S. aureus*. Control tubes inoculating 5 mL of NB only with *S. aureus* were made. Tubes were incubated at 35 °C in an orbital shaker and evaluated for the growth of *S. aureus* after 24, 48, and 72 h by seeding 100 µL of each solution in Mannitol Salt Agar (MSA, Oxoid, Basingstoke, UK) and the growth after 24 h of incubation at 35 °C was evaluated.

The MIC was defined as the lowest concentration of the compound or drug that inhibits the growth of a microorganism, and it is expressed as mg/mL or μg/mL; the MBC was defined as the lowest concentration of the extract that results in killing 99.9% of the tested bacteria [27]. Each experiment was performed in triplicate.

### 2.4. Experimental Cheese Preparation

Experimental cheese samples were prepared by using fresh pasteurized whole milk produced by “Parmalat, Stabilimento di Catania” (Italy). Milk bottles (1 L each) were bought in a local supermarket on their production date, coincident with the first day they were available to consumers, transported to the laboratories of Di3A (University of Catania) under refrigerated conditions, and immediately used for cheese preparation. The nutritional label of the milk used reports the following information: fat, 3.60 g/100 mL; carbohydrates, 4.9 g/100 mL; protein, 3.4 g/100 mL; and salt, 0.13 g/100 mL. Each experimental cheese, supplemented with the two extracts described above, was prepared following the protocol reported by Han et al. [28] with slight modifications. In brief, calcium chloride (CaCl_2_) was added to milk to achieve a final concentration of 6 mM. Afterwards, each lyophilized extract was added to the milk at a concentration of 0.5 mg/mL. The control sample was made without any extract addition. Milk containing only CaCl_2_ had a pH value of 6.50, while both milk samples that contained the PPW or PMW extract registered a pH of 6.46. A commercial calf liquid rennet solution (Vik Cheese, Beer & Wine, Parma, Italy) was added to each milk sample to obtain a concentration of 5% *v/v*. The samples were then heated in a water bath at 35 °C for 2 h. After coagulation, the samples were centrifuged at 1300× *g* at 21 °C for 15 min in a 4239R ALC centrifuge (ALC, Winchester, VA, USA); the whey (serum) was separated from the curd (gel) and the latter was put in trays of 4 × 4 cm with a depth of 3 cm. All samples were stored for 5 h at 4 ± 1 °C for later experiments. Cheese samples under study are reported in Table 1, and the preparation procedure is displayed in Figure S1.

#### 2.4.1. Polyphenol Content of Cheese

In order to recover pomegranate phenolics from cheese curd, an aliquot (5 g) of each sample under study (Table 1) was extracted with a mixture of methanol:formic acid:water (80:1:19), filtered, and centrifuged twice at 15 °C and 9000 rpm (Sorvall RC-5B Refrigerated Super Speed centrifuge, Fisher Scientific Italia, Rodano (MI), Italy). The clear supernatants were transferred to 2 mL HPLC amber vials and immediately analyzed according to Section 2.2.

#### 2.4.2. Estimated Cheese Yield, pH, and Titratable Acidity

The estimated cheese yield, defined as the quantity of milk needed to produce 1 kg of cheese, was calculated for each sample of experimental cheese under examination (Table 1) and expressed as a yield percentage (Y%) using the following formula:

Y% = (cheese weight/milk weight) × 100(1)

The difference in the yield of the cheese supplemented with extract (cheese-PPW or cheese-PMW) and of the control sample was evaluated by comparing the mean yield % value obtained from three independent replications.

The pH and titratable acidity variation of cheese either supplemented or not with the pomegranate extracts were assessed according to the standard methods described by the Association of Official Analytical Chemists [29].

#### 2.4.3. Color and Texture

The color of all samples was described in terms of lightness (L*), redness (a*), and yellowness (b*) space values (CIE L* a* b*). The measurements were carried out on two different points of the cheese surface exposed to air by using a Konica Minolta CM-2500d (Konica Minolta sensing Europe B.V., Bremen, Germany). The color difference between the control sample and samples containing the pomegranate extract (cheese-PPW or cheese-PMW) was expressed as the mean value ± standard deviation of six readings. Each cheese sample was produced in triplicate in order in to achieve a total of six readings for each sample.

Cheese samples, prepared as previously described, were analyzed for their textural properties by using a Texture Analyzer Zwick/Roell model Z010 (Zwick Roell Italia S.r.l., Genova, Italy), equipped with a cylindrical probe of 2.5 cm in diameter, following the method reported by Gutiérrez-Méndez et al. (2013) [30] with minor modifications.

Cheese samples were portioned into squares (2 cm × 2 cm) and compressed at 75% using a pre-load of 0.01 N, a cell load of 50 N, and a cross-head speed of 0.05 cm/s. Replications were conducted using each time a new square not subject to a previous compression. The hardness (N) of each sample, representing the maximum force (Fmax) required to reach the point of break, was expressed as the mean ± standard deviation; the test was repeated three times.

### 2.5. In Vivo Bactericidal Activity towards Induced S. aureus Contamination of Cheese

The antibacterial activity of PPW and PMW was evaluated against *S. aureus* inoculated in milk in order to simulate cheese contamination.

Briefly, a single colony of *S. aureus* was inoculated into Tryptone Soy broth (Oxoid, Basingstoke, UK) and shaken overnight to prepare a working solution. The concentration of the bacterial suspension was adjusted to 10^9^ CFU/mL in a sterilized 0.9% sodium chloride (NaCl) solution. An adequate quantity of bacterial suspension was centrifuged, and the obtained pellet was washed twice with sterile distilled water and added to milk to obtain an initial concentration of 10^5^ CFU/mL.

The inoculated milk was used to produce the experimental cheese following the procedure described in the Section 2.4. Cheese samples, both supplemented or not with the pomegranate extracts (cheese-PPW and cheese-PMW), were sealed, placed at 4 °C, and analyzed on the same day of production as well as after 1, 2, 4, 8, and 12 d of refrigerated storage. At each sampling time, cheese samples, contaminated or not with *S. aureus*, were aseptically transferred into a Stomacher bag containing a proportional amount of sterile Ringers solution (Oxoid, Basingstoke, UK) and homogenized for 5 min. Serial decimal dilutions of the obtained suspension were prepared with the same diluent. The growth of *S. aureus*, in contaminated cheese, was evaluated by spread-plating 0.1 mL of appropriate dilutions on Mannitol Salt Agar (MSA Oxoid, Basingstoke, UK) and incubation at 32 °C for 24 h. Cheese samples not inoculated with *S. aureus* was analyzed for total mesophilic bacteria (TMB), *Enterobacteriaceae*, and *Lactobacillus* spp. count, using Plate Count Agar (PCA, CM325, Oxoid, Basingstoke, UK), Violet Red Bile Glucose Agar (VRBGA, CM0485, Oxoid, Basingstoke, UK), and MRS Agar, respectively, and incubated at 32 °C for 24–48 h. Each microbiological count was performed in triplicate and expressed as log_10_ CFU/g of cheese.

### 2.6. Statistical Analysis

Data from in vitro and in vivo experiments were analyzed separately by using the Statistical package software Minitab ™ version 16.0. A two-way analysis of variance (ANOVA) was carried out to determine the significance (*p* < 0.05) of the main effects (treatment and storage time) on the growth of *S. aureus*. One-way analysis of variance (ANOVA) was performed on mean values and Fisher’s test was carried out for the comparison of difference among treatments. Differences between sample means were considered significant at *p* ≤ 0.05. Standard deviation was obtained from the statistical model and is shown as bars in the figures.

## 3. Results and Discussion

### 3.1. Identification and Quantifications of Polyphenols in Pomegranate Byproduct Extracts

Pomegranate fruit is characterized by the presence of polyphenols, mainly hydrolyzable tannins, in considerable amounts.

Gallotannins and ellagitannins are the most represented subclass of polyphenols, followed by simple ellagic acid derivatives and anthocyanins; nevertheless, the peculiarity of this matrix is undoubtedly the presence of the anomers punicalagin a and b [18]. The secondary metabolic profile and content of peel and mesocarp from local pomegranate fruits were studied in the present work, employing a series of HPLC/UV-Vis-DAD and HPLC/ESI-MS analyses. The corresponding DAD chromatograms, visualized at 280 nm, are shown in Figure S2 (supplementary materials). Eighteen peaks were detected and tentatively identified basing on their relative retention times, UV-Vis, mass spectral data, and injection with pure analytical standards when available. Comparison with literature data further corroborated the assignments, reported in Table 2.

The UV-Vis spectra of the detected peaks allowed for the distinguishing of gallotannins (peaks number 1, 6, 9, 11, 13, and 14, for a total of six) from molecules bearing at least one ellagic acid moiety, which can be further divided into ellagitannins (six peaks: 2, 3, 4, 5, 7, and 8) and ellagic acid simple derivatives (peaks number 10, 12, 15, 16, 17, and 18). Molecules within these subclasses share almost identical UV-Vis spectra, and only the contribution of mass spectrometric data derived from TICs (total ion current chromatograms) and EICs (extracted ion chromatograms) made a rational identification possible. The extracts under investigation resulted in being very dissimilar, both qualitatively and quantitatively. Under a qualitative point of view, the water extract from pomegranate mesocarp (PMW) is characterized by the presence of punicalagins (peaks 7 and 8) as the main compounds, whilst that obtained from pomegranate peel (PPW) is clearly dominated by the ellagitannin punicalin and its isomer (peaks 3 and 4). Another substantial difference is given by ellagic acid (peak 18), present as a minor compound in PMW while being the third most abundant metabolite in PPW. PPW is also ca. four times richer in polyphenols to respect to PMW (Table 2), as broadly reported in the literature [18].

### 3.2. In Vitro Antibacterial Activity of Pomegranate Extracts

#### 3.2.1. Antibacterial Activity In Vitro against Target Pathogens

Table 3 reports the antibacterial activity of pomegranate extracts performed on NA by the agar well diffusion assay. The two lyophilized pomegranate extracts were diluted in sterile distilled water as reported in the Section 2, and then tested against the target bacteria mentioned above.

Both extracts evidenced a good antibacterial activity against *S. aureus*, *S. hemolyticus*, *P. fluorescents*, *B. subtilis*, and *B. cereus*, giving rise to an inhibition zone equal or wider than 0.50 cm.

Although both extracts were active against the same strains, PPW showed an average inhibitory activity significantly higher than that registered by PMW (Table 3). No antimicrobial activity was detected towards *L. monocytogenes*, *L. innocua*, *L. gray*, and *S. enterica*. Moreover, despite the fact that different authors [31,32,33] report that pomegranate peel extract can inhibit the growth of *E. coli*, in the present study no antimicrobial activity was observed towards this species. Controversial results can be attributed to the different extraction method used or could depend on several factors, such as the cultivar, seasonality, and geographical origin of the fruits [34,35]. The different sensitivity of tested bacteria to PPW and PMW extracts is probably due to the variable composition of the various parts of the plant used to produce each extract, and consequently to the different content of bioactive compounds in the two extracts.

#### 3.2.2. In Vitro Antimicrobial Activity of Pomegranate Extracts against *S. aureus*

**Figure 1:**
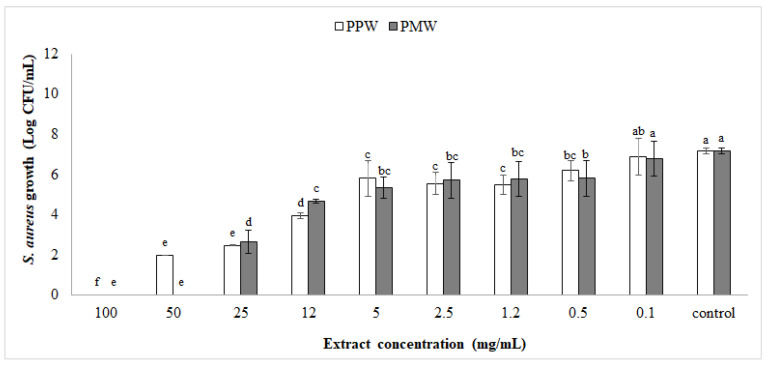
In vitro antimicrobial activity of pomegranate extracts from peel (PPW) or mesocarp (PMW) against *S. aureus*. Vertical bars indicate the standard deviation of the mean. Values in columns marked by a different letter are significantly different according to Fisher’s least significant difference test (*p* < 0.05).

Figure 1 shows the in vitro antibacterial activity of different extract concentrations towards *S. aureus.* All the tested concentrations, with the exception of 0.1 mg/mL, significantly (*p* < 0.05) reduced the growth of *S. aureus* in comparison to the control sample without the extract, which reached the highest growth value (7.17 ± 0.1 log CFU/mL).

For both extracts, PPW and PMW, the lowest concentration that gave rise to a significant (*p* < 0.05) inhibition of the growth of *S. aureus* (MIC) was 0.5 mg/mL. The growth of *S. aureus* in broth containing 0.5 mg/mL of extract was at least one logarithmic unit lower than the control sample (0.00 mg/mL), registering values of 6.19 ± 0.51 log CFU/mL and 5.81 ± 0.88 log CFU/mL, respectively, for PPW and PMW. For both extracts, PPW and PMW, the concentration that gave rise to a 99.9% reduction in bacteria growth (MBC), in comparison to the control, was equal to 25 mg/mL. Although different authors [36,37,38] report that pomegranate peel extract has the greatest antimicrobial activity in comparison to extract obtained from seed, fruit, and juice, in the present study the extract obtained from mesocarp (PMW) evidenced antimicrobial activity in vitro towards *S. aureus* comparable, or even higher, to that of the extract obtained from peel (PPW), especially when employed at a high concentration. In our study, the MICs of pomegranate extracts were approximately ten time higher than that reported by Nozohour et al. [38] for a pomegranate ethanolic extract made from peel or seed against *S. aureus*, while the same MBCs were recorded.

### 3.3. Evaluation of Cheese Samples Containing Pomegranate Extract

#### 3.3.1. Polyphenol Content

Table 4 displays the amount of polyphenols identified from cheese extraction (see also Figure S3). Cheese–PPW evidenced the highest content of total polyphenols in comparison to cheese–PMW. In cheese–PPW, ellagic acid (peak 18) was the most representative polyphenol (0.0742 mg/g), followed by ellaggitannins (peaks 3 and 4). In cheese-PMW, ellagic acid (18) concentration was almost five-fold lower than cheese-PPW (registering a value of 0.0140 mg/g). Only punicalagins (7 and 8) are present in a relative high concentration in cheese-PMW compared to cheese-PPW (not quantified). These results showed that the differences registered in extract composition (Table 2) are kept during cheese formation (Table 4), with no evidence of any preferential retention from this matrix.

#### 3.3.2. Estimated Cheese Yield, pH, and Titratable Acidity

Table 5 reports the yield, pH, and titratable acidity of the cheese samples under study. Cheese–PPW evidenced the highest value of yield % (65.36 ± 0.32%). Insignificant (*p* > 0.05) differences were observed between the yield % of cheese-PPW and the control. The yield of cheese-PMW was significantly (*p* < 0.05) lower than that of the control, registering a value of 61.56 ± 0.39%. The addition of pomegranate extracts affected the pH of cheese compared to the control (6.32 ± 0.050), registering the significantly (*p* < 0.05) highest values of 6.52 ± 0.035 and 6.53 ± 0.005, respectively, for cheese-PPW and cheese-PMW. In comparison to the control, the significantly (*p* < 0.05) lowest value of titratable acidity was detected in the cheese-PPW sample. In contrast with our results, Mahajan et al. [20] concluded that the use of rind pomegranate extract, as a dipping solution, can strongly influence the pH of Kalari cheese, determining significantly (*p* < 0.05) lower values compared to the control. These different results are probably due to the application method and to the highest concentration of the extract employed (1% and 2%). Moreover, other authors reported that low-fat cheese supplemented with plant extract or essential oils, rich in polyphenols, always registered a low pH value in comparison to the control [39,40].

#### 3.3.3. Color and Texture

As reported in Table 6, the addition of the extracts PPW and PMW influenced the color of the cheese, determining a decrease in L* and a* values and an increase in the value of b* (see also Figure S4).

The color change of cheese samples is certainly due to the bond that the phenolic compounds created with milk during the cheese-making process, more evident in the cheese-PPW sample that registered the lowest L* and a* values and the highest b* and C parameters. As seen from previous tests, in fact, cheese-PPW is the sample that contains the highest content of total polyphenols (Table 4). Probably due to the different application method, our results are not in accordance with those reported by Mahajan et al. (2015) [25], who showed that the use of pomegranate rind extract as a dipping solution did not determine a significant (*p* > 0.05) change in the color of extract-added cheese compared to the control. The use of PPW and PMW extracts, in a concentration of 0.5 mg/mL, determined a modification of the textural parameters; in fact, cheese-PPW and cheese-PMW evidenced significantly (*p* < 0.05) higher hardness values compared to the control. This result is in contrast with that reported by Han et al. (2011) [41], who evidenced that the addition of phenolic compounds, in a concentration equal to that used in the present study, did not affect cheese firmness.

### 3.4. Antimicrobial Activity of Pomegranate Extracts Incorporated into Experimental Cheese

**Figure 2:**
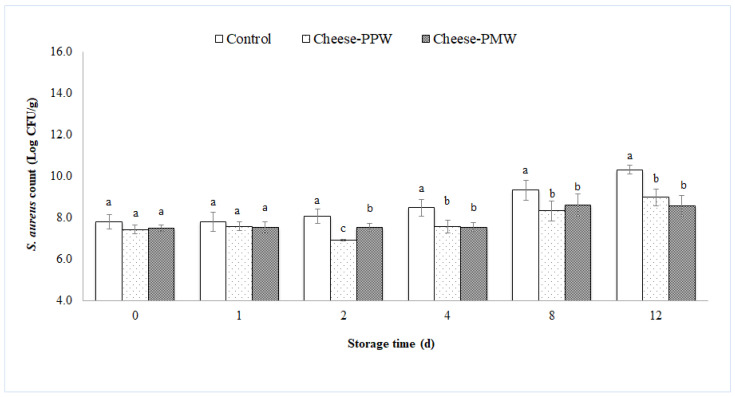
Growth of *S. aureus*, evaluated up to 12 days of storage at 4 ± 1 °C on cheese supplemented with pomegranate extracts (cheese-PPW and cheese-PMW). Columns at the same storage time (0, 2, 4, and 8 days) followed by different letters are significantly different according to Fisher’s least significant difference test (*p* < 0.05). Vertical bars indicate the standard deviation of the mean.

Cheese samples containing *S. aureus* were prepared using pasteurized milk intentionally contaminated with *S. aureus* at a concentration of 6.79 ± 0.57 log CFU/mL. After the cheese-making process (day 0), the growth of *S. aureus* in cheese reached a concentration of 7.8 ± 0.35 log CFU/g, 7.4 ± 0.20 log CFU/g, and 7.5 ± 0.17 log CFU/g, respectively, for the control sample, the sample containing PPW, and the sample containing PMW (Figure 2). A two-way ANOVA indicated that both main effects (treatment and storage time) were significant (*p* < 0.05), and that there was a significant interaction between treatment and storage time. The effect of the addition of the two extracts to the experimental cheese significantly (*p* < 0.05) affected the *S. aureus* count, which, starting from 24 h after the cheese preparation to the last day of refrigerated storage (12 d), was averagely reduced by more than one logarithmic unit, reaching values of 8.97 ± 0.39 log CFU/g in cheese-PPW and 8.57 ± 0.49 log CFU/g for cheese-PMW, compared to the control, which reached a value of 10.30 ± 0.21 log CFU/g (Figure 2).

This result is particularly worthy of note, considering that the concentrations of the extracts added to milk during the cheese-making process were those of the MICs, calculated in vitro, and that due to the complexity of food matrices rich in fat and proteins, such as cheese, natural antimicrobial compounds can be made less effective by binding with some food components. Smith-Palmer et al. (2001) [42] concluded that the chemical composition of cheese was a key factor in the antimicrobial efficacy of some natural compounds, since only in low-fat cheese were all the essential oils under study (bay, clove, cinnamon, and thyme) effective in limiting the growth of *Listeria monocytogenes*, while in cheese with a high fat content only clove essential oil showed a comparable activity. Similarly, Gutierrez et al. (2008) [43] reported that the antimicrobial activities of oregano and thyme essential oils against *L. monocytogenes* were decreased by high lipid levels in a simulated food matrix. Thus, to obtain the same inhibitory capacity observed in vitro, the same natural compound should be added to foods at higher concentrations [44]. Moreover, natural bioactives can be lost during cheese making due to their higher solubility in the whey [45] or to their sensibility to food intrinsic and extrinsic factors [46]. This was also affirmed by Gammariello et al. (2008) [47], who observed that higher concentrations of the tested natural compounds were needed to achieve the same antimicrobial effect in Fior di latte cheese, than that tested in vitro. Similarly, the concentration of essential oil extracted from pink pepper tree, equivalent to the MIC against *L. monocytogenes* calculated in vitro, was not equally effective toward the same pathogen in the Minas-type fresh cheese [48].

Cheese prepared with pasteurized milk not inoculated with *S. aureus* evidenced a total *Enterobacteriaceae* and *Lactobacillus* spp. count below the detection limit of the plate count method throughout the considered storage period of 12 d. The absence of such a microbial group can be attributed to the thermal treatment of milk and to the hygienic practices followed during the preparation and packaging of experimental cheese samples.

**Figure 3:**
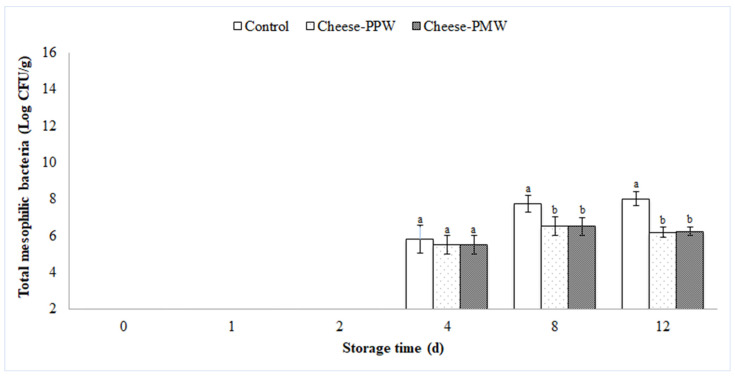
Growth of total mesophilic bacteria (TMB), evaluated up to 12 days of storage at 4 ± 1 °C on control cheese and cheese incorporating pomegranate extracts (cheese–PPW and cheese–PMW). Columns at the same storage time (0, 2, 4, and 8 days) followed by different letters are significantly different according to Fisher’s least significant difference test (*p* < 0.05). Vertical bars indicate the standard deviation of the mean.

The TMB count, displayed in Figure 3, was below the detection limit up to 2 d of storage, whereas after 4 d of storage an increase in bacterial growth was detected in all samples, with values of 5.84 ± 0.77 log CFU/g, 5.50 ± 0.50 log CFU/g, and 5.50 ± 0.50 log CFU/g, respectively in the control, cheese–PPW and cheese–PMW samples. After 8 d of storage, a statistically significant (*p* < 0.05) difference in TMB count was detected between the control and cheese-PPW and cheese-PMW samples; the latter two, in fact, recorded a TMB value of 6.51 ± 0.51 log CFU/g and 6.52 ± 0.49 log CFU/g, respectively, almost one logarithmic unit lower than the control (7.75 ± 0.47 log CFU/g). The same trend was also confirmed after 12 d of storage, where the TMB counts remained almost constant in cheese-PPW and cheese-PMW (6.20 ± 0.27 log CFU/g and 6.25 ± 0.25 log CFU/g, respectively), while the control sample registered the significantly (*p* < 0.05) highest TMB value of 8.02 ± 0.37 log CFU/g. Such results are in accordance with the study of Mahajan et al. (2015) [25], who confirmed that the addition of a commercial pomegranate rind extract at 1 and 2% to Kalari cheese significantly reduced total plate count with respect to the control cheese throughout the storage (14 d).

## 4. Conclusions

The present work evaluated the suitability of two extracts obtained from pomegranate peel and mesocarp to be used as natural food preservatives, which are the most quantitatively representative byproducts of pomegranate processing. Pomegranate extracts, PPW and PMW, showed a strong antibacterial activity in vitro against different target bacterial strains, among which *S. aureus* was selected for further in vivo assays in an artificially contaminated cheese matrix. The HPLC analysis revealed that, between the two extracts, PPW is the one that contains the highest amount of ellagic acid, which is generally considered the major factor responsible for the greater antimicrobial activity of the pomegranate extract [49]. After the cheese-making process, the PPW determined a greater change in the color of cheese but it did not affect the estimated cheese yield or titratable acidity in comparison to the control. Furthermore, both extracts determined an increase in firmness if compared to the control. As revealed by the HPLC analysis, cheese-PPW contained the highest amount of ellagic acid and total polyphenols compared to cheese-PMW. Despite this difference, both cheese samples evidenced, after 12 d of conservation, a one logarithmic unit decrease in *S. aureus* and TBM growth when compared to the control, suggesting that the antimicrobial activity of the two extracts is not directly correlated with the ellagic acid amount, but is related to the synergism of bioactive compounds.

Such results, though preliminary, would encourage further scientific research aimed at the reuse of agro-food byproducts, as a substitution for chemical food preservatives, as the key to a circular economy.

## Figures and Tables

**Table 1:** Formulations of cheese samples under study.

Sample Code	Cheese Ingredients
Control	Milk + 0.066% (*w/v*) CaCl + 5% (*v/v*) rennet
Cheese-PPW	Milk + 0.066% (*w/v*) CaCl + 5% (*v/v*) rennet + 0.05% (*w/v*) PPW
Cheese-PMW	Milk + 0.066% (*w/v*) CaCl + 5% (*v/v*) rennet + 0.05% (*w/v*) PMW

**Table 2:** Total polyphenol amount, as measured by HPLC, in the pomegranate byproduct matrices analyzed in this study. Results are reported as mg of compound per gram of fresh vegetable material and as the mean of three replicates. PPW = pomegranate peel water extract; PMW = pomegranate mesocarp water extract; n.d. = not detected. See text for further details.

Peak No.	Compound Identified	PPW, mg/g of Fresh Vegetable Material	PMW, mg/g of Fresh Vegetable Material
1	Galloyl hexoside	n.d.	0.081
2	Punicalagin derivative	0.609	0.347
3	Punicalin (gallagyl-hexoside)	8.437	0.242
4	Punicalin isomer	10.246	0.029
5	Punicalagin isomer	1.084	0.106
6	Digalloyl-hexoside	0.051	n.d.
7	Punicalagin a	0.155	1.853
8	Punicalagin b	0.643	3.801
9	Punigluconin isomer	1.341	n.d.
10	Ellagic acid deoxy-hexoside	n.d.	0.169
11	Pedunculagin isomer	0.309	n.d.
12	Ellagic acid hexoside	2.217	0.724
13	Granatin B	0.127	n.d.
14	Granatin B isomer	0.230	n.d.
15	Ellagic acid galloyl-hexoside	0.101	0.042
16	Ellagic acid pentoside	0.765	0.089
17	Ellagic acid galloyl-hexoside isomer	1.051	0.101
18	Ellagic acid	6.679	0.242
	Total polyphenol	34.045	7.826

**Table 3:** In vitro antimicrobial activity of pomegranate peel water (PPW) and pomegranate mesocarp water (PMW) extracts against different bacterial strains. - = no inhibition halo.

Bacterial Species	Inhibition Halos (diam., mm)	
	PPW	PMW
*S. aureus*	7.7 ± 0.6a	5.0 ± 0.0b
*S. hemolyticus*	8.3 ± 0.6a	5.3 ± 0.6b
*P. fluorescens*	9.7 ± 0.6a	7.0 ± 1.0b
*B. subtilis*	9.3 ± 0.6a	6.0 ± 1.0b
*B. cereus*	9.7 ± 0.6a	7.3 ± 0.6b
*L. monocytogenes*	-	-
*L. innocua*	-	-
*L. gray*	-	-
*S. enterica*	-	-
*E. coli*	-	-

Data presented as mean ± standard deviation of three independent replicates. In each row, values followed by a different letter are significantly different according to Fisher’s least significant difference test (*p* < 0.05).

**Table 4:** Polyphenol content in experimental pomegranate-enriched cheese samples. n.q. = not quantified.

Peak No.	Compound Tentatively Identified	Cheese–PMW, mg/g	Cheese–PPW, mg/g
3	Punicalin (gallagyl-hexoside)	0.0027	0.0159
4	Punicalin isomer	0.0022	0.0176
7	Punicalagin a	0.0108	n.q.
8	Punicalagin b	0.0156	n.q.
12	Ellagic acid hexoside	0.0121	0.0145
17	Ellagic acid galloyl-hexoside isomer	0.0015	0.0064
18	Ellagic acid	0.0140	0.0742
	Total polyphenols	0.0588	0.1286

**Table 5:** Yield and physico-chemical properties of cheese containing pomegranate extracts.

Sample	Estimated Cheese Yield, %	pH	Titratable Acidity, %
Control	64.72 ± 0.45 a	6.32 ± 0.050 b	0.025 ± 0.002 a
Cheese–PPW	65.36 ± 0.32 a	6.52 ± 0.035 a	0.023 ± 0.000 ab
Cheese–PMW	61.56 ± 0.39 b	6.53 ± 0.005 a	0.020 ± 0.002 b

Data presented as mean ± standard deviation of the mean. Values in columns followed by different letters within the same parameter are significantly different according to Fisher’s least significant difference test (*p* < 0.05).

**Table 6:** Color and texture of cheese samples enriched with pomegranate extracts.

Samples	Color Parameter				Texture Parameter
	L*	a*	b*	C	Hardness (N)
Control	95.72 ± 0.07 a	−1.94 ± 0.01 a	8.40± 0.03 c	8.62 ± 0.03 c	0.50 ± 0.02 b
Cheese-PPW	90.20 ± 0.90 c	−3.50 ± 0.14 c	19.9 ± 0.52 a	20.24 ± 0.54 a	0.56 ± 0.02 a
Cheese-PMW	92.00 ± 0.43 b	−2.20 ± 0.11 b	13.8 ± 0.64 b	13.98 ± 0.64 b	0.53 ± 0.00 a

Data presented as mean ± standard deviation of the mean. Values in columns followed by a different letter within the same parameter (L*: lightness; a*: redness; b*: yellowness; C: chroma; and hardness) are significantly different according to Fisher’s least significant difference test (*p* < 0.05).

## Data Availability

The data presented in this study are available on request from the corresponding author.

## Supplementary Materials

The following are available online at https://www.mdpi.com/article/10.3390/foods10112669/s1, Figure S1: Experimental procedure used for cheese preparation. Figure S2: HPLC-DAD chromatograms, visualized at 280 nm, of the pomegranate byproduct extracts analyzed in this study: PMW, pomegranate mesocarp water extract (above) and PPW, pomegranate peel water extract (below). For peak assignments, see Table 2 and text. n.i. = not identified (unclear UV–Vis and troublesome mass spectrum). Figure S3: HPLC-DAD chromatograms, visualized at 360 nm, of the cheese matrices analyzed in this study (blue and pink line) plus the control (black line). See also Table 4 and text for further details. Figure S4: Cheese samples immediately after coagulation phase (on the left) and after mold release (on the right): (a) control (without any extract addition), (b) cheese supplemented with pomegranate peel water extract and (c) cheese supplemented with pomegranate mesocarp water extract.
