# Supplementary figures and images for: Pomegranate Byproduct Extracts as Ingredients for Producing Experimental Cheese with Enhanced Microbiological, Functional, and Physical Characteristics
Source: Foods. 2021 Nov 3;10(11):2669. doi: 10.3390/foods10112669 (PMC8621625; doi:10.3390/foods10112669)

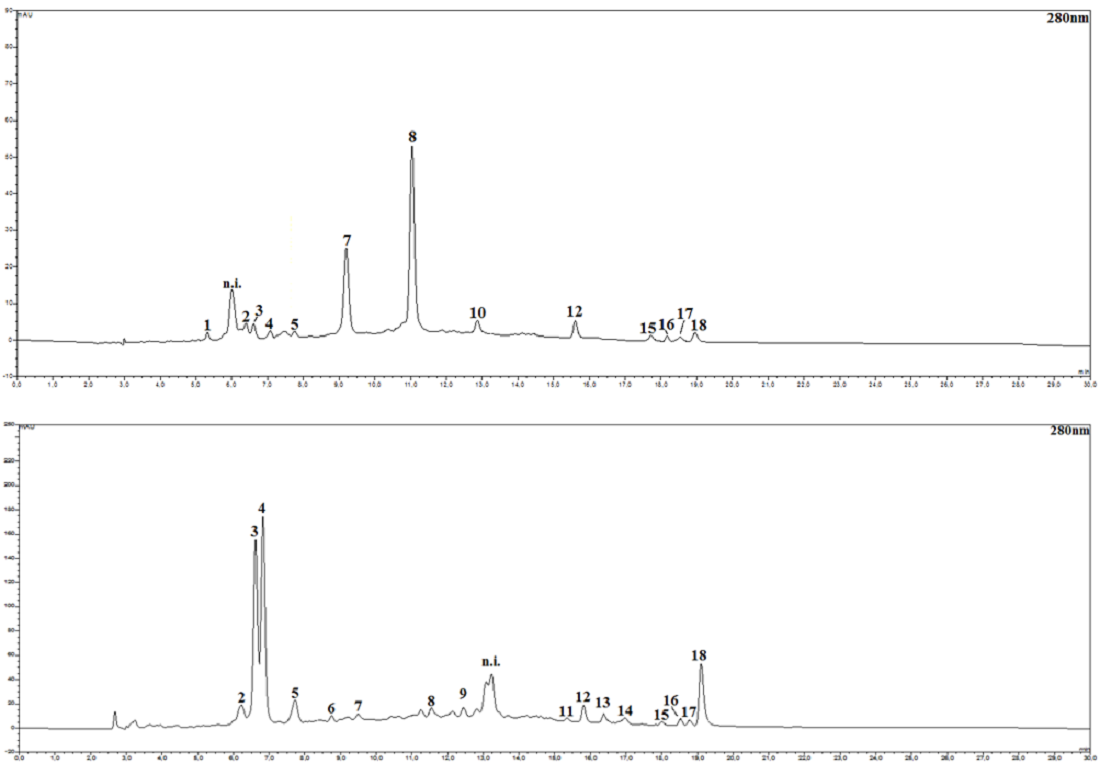

Supplement: Supplementary file 1 [file foods-10-02669-s001.zip › Figure S2.tif]

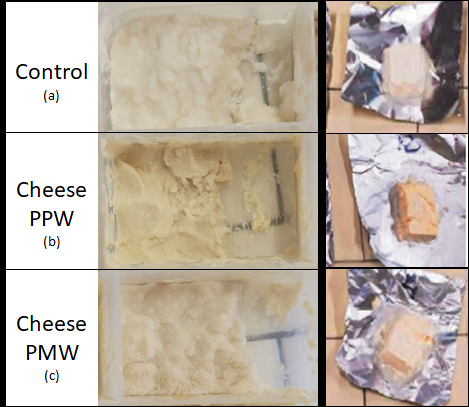

Supplement: Supplementary file 1 [file foods-10-02669-s001.zip › Figure S4.tif]

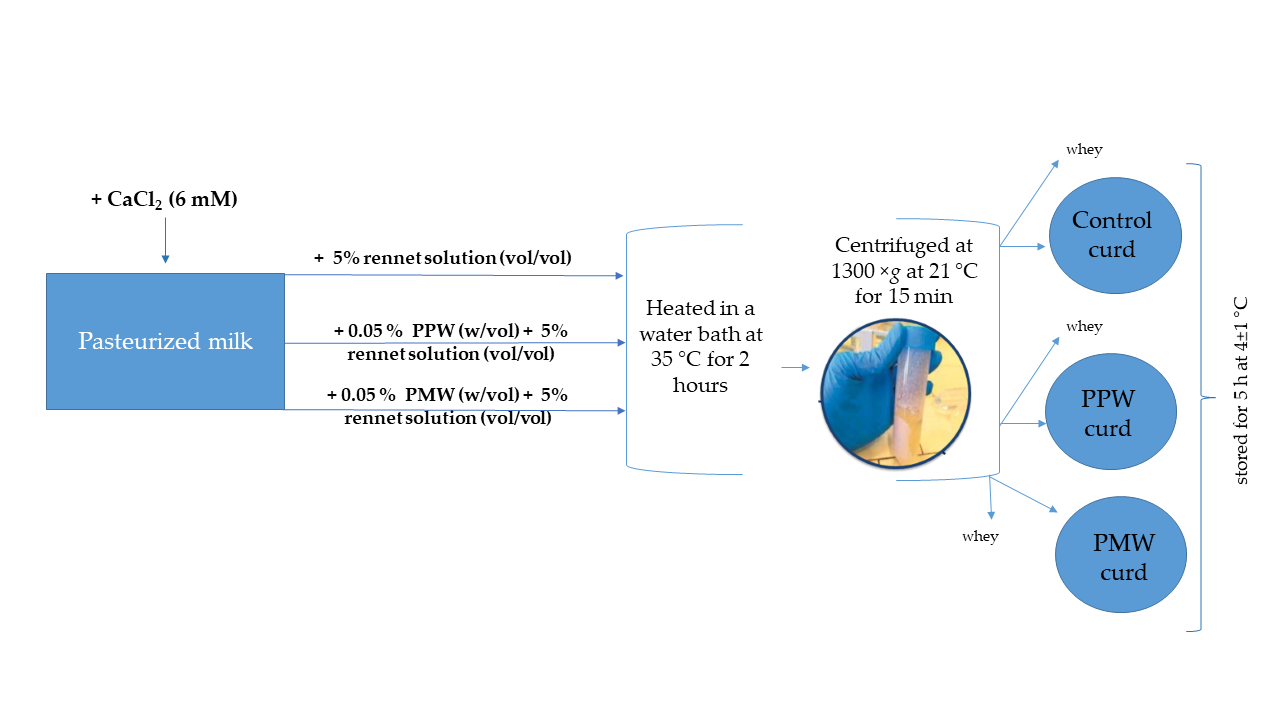

Supplement: Supplementary file 1 [file foods-10-02669-s001.zip › Fig. S1.tif]
